# Pinocembrin, an anticancer dihydroxyflavanone: from chemistry to cellular interactions and synergistic prospects

**DOI:** 10.1186/s12935-026-04352-w

**Published:** 2026-06-12

**Authors:** Renu Chane, Shallu Saini, Rohit Bhatia, Katrin Sak, Isha Rani, Shafiul Haque, Adesh Kumar Saini, Reena V. Saini, Hardeep Singh Tuli, Arif Hussain

**Affiliations:** 1 Department of Biochemistry, Sharda School of Medical Sciences and Research, Sharda University, Greater Noida, Uttar Pradesh India; 2 Department of Bio-Sciences and Technology, Maharishi Markandeshwar Engineering College, Maharishi Markandeshwar (Deemed to Be University), Mullana, Ambala, 133207 India; 3 Chitkara College of Pharmacy, Chitkara University, Rajpura, Punjab India; 4 NGO Praeventio, Tartu, Estonia; 5 Department of Biochemistry, RIMT Medical College and Hospital , Mandi-Gobindgarh , Punjab 147301 India; 6 Department of Nursing, College of Nursing and Health Sciences, and Health Research Center, Jazan University, 82911 Jazan, Saudi Arabia; 7 School of Medicine, Universidad Espiritu Santo, Samborondon, 091952 Ecuador; 8 Manipal Institute of Health & Life Sciences, Manipal Academy of Higher Education, Dubai Campus, G04, P.O Box 345050, Dubai, United Arab Emirates

**Keywords:** Dihydroxy flavanone, Apoptosis, Cell cycle, Anti-proliferative, Synergistic

## Abstract

Cancer remains a major global health challenge, with rising incidence and persistent therapy resistance highlighting the need for new multi-targeted therapeutic agents. Pinocembrin (5,7-dihydroxyflavanone), a naturally occurring flavanone found in honey, propolis, and several medicinal plants, has gained increasing attention for its broad-spectrum anticancer potential. Recent studies demonstrate that pinocembrin modulates multiple hallmarks of cancer by regulating the PI3K/Akt/mTOR, STAT3, and NF-κB pathways, resulting in antiproliferative, pro-apoptotic, anti-metastatic, and anti-inflammatory effects. Evidence from diverse cancer models—including breast, prostate, colon, lung, ovarian, and melanoma—shows that pinocembrin induces cell-cycle arrest, activates intrinsic and extrinsic apoptotic pathways, inhibits angiogenesis, and suppresses epithelial–mesenchymal transition. Structure–activity relationship (SAR) analyses further reveal that modifications such as hydroxylation, esterification, and glycosylation enhance its bioavailability and anticancer activity. Despite its therapeutic promise, the clinical utility of pinocembrin is limited by poor solubility and rapid metabolic clearance. Recent nanotechnology-based formulations, including polymeric micelles, liposomes, nanoparticles, and nanoemulsions, have significantly improved their stability, bioavailability, and tumor-targeted delivery. Pinocembrin also exhibits synergistic effects with conventional chemotherapeutics while maintaining low toxicity toward normal cells, underscoring its suitability for combination therapy and chemoprevention. Preliminary clinical data indicate a favorable safety profile, although long-term toxicity, optimal dosing, and pharmacokinetic parameters require further investigation. This review synthesizes current knowledge on the anticancer mechanisms, SAR-driven insights, nanotechnology-enhanced delivery, synergistic actions, and safety considerations of pinocembrin. By integrating recent findings and highlighting research gaps, it provides a comprehensive foundation for advancing pinocembrin toward future preclinical and clinical applications in oncology.

**Graphical Abstract:**

**Figure:**
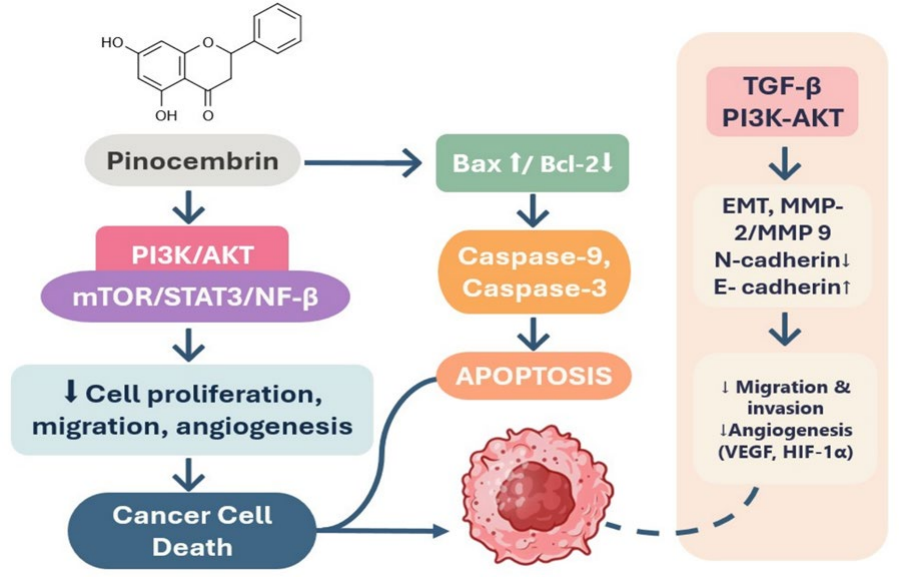

## Introduction

Cancer has remained one of the most dreadful diagnoses. There were almost 20 million new cancer cases and 9.7 million cancer deaths in the year 2022, whereas the global cancer incidence is expected to rise to 35 million, i.e., 77%, by 2050. Therefore, novel therapeutic strategies are highly needed to combat the steadily increasing cancer burden all over the world. These findings underscore the urgent need to discover novel, multi-functional agents that can bypass or overcome these resistance mechanisms [1]. To date, a considerable part of the clinically approved cancer drugs has been initially derived from diverse natural sources, including various higher plants. Such medications include vincristine, vinblastine, and paclitaxel. However, only about a tenth of all higher plants have been investigated for the possible anticancer potential of their constituents, so any effort to identify novel molecular entities that would be safe and efficient in preventing or treating cancer remains critical [2]. Plant-derived bioactive compounds offer significant chemopreventive potential by inhibiting or delaying cancer development. Because of their unmatched structural variety, intricate architecture, and evolutionary optimization for biological interactions, phytochemicals are essential in the drug development process [3, 4]. When compared to synthetic compounds, they frequently offer greater efficacy and unique methods of action, making them a better option for managing cancer. Flavonoids, terpenoids, alkaloids, and phenolic acids can modulate key processes in tumor initiation and progression, including oxidative stress, inflammation, apoptosis, and cell cycle regulation (Tumbarski et al., 2025; [5–7]). Flavonoids, in particular, act on multiple signaling pathways such as Nuclear Factor kappa-light-chain-enhancer of activated B cells (NF-κB), Mitogen-activated protein kinase (MAPK), and phosphatidylinositol 3-kinase and protein kinase B (PI3K/Akt), contributing to their broad therapeutic relevance [8]. Due to their low toxicity and multi-targeted effects, they are promising candidates for long-term prevention strategies. Pinocembrin, as a flavonoid, exemplifies this potential through its anti-inflammatory, antioxidant, and antiproliferative actions in various cancer models.

One phytochemical that has recently gained more scientific attention for its potential anticancer properties is pinocembrin (5,7-dihydroxyflavanone). This low-molecular-weight polyphenolic compound belongs to the flavanones subclass of the flavonoid family and can be found in many natural sources, such as damiana, fingerroot, honey, and propolis [9, 10]. Several preclinical studies have revealed multiple biological activities of this phytochemical, exerting antioxidant, anti-inflammatory, antimicrobial, and neuroprotective but also anticancer effects [9, 11]. Indeed, pinocembrin has been demonstrated to suppress the development of malignant cells derived from the human lung, colon, liver, breast, ovary, prostate, and skin through cell cycle arrest, apoptotic induction, restricting angiogenic responses, and disrupting metastatic spread [12–17]. Various molecular targets have been described for this compound, intervening in multiple cancer-related signaling processes [18]. Research findings provide advanced knowledge regarding the complex characteristics of autophagy in cancer therapy and pharmaceutical resistance [19]. The research by Yang et al. [20] demonstrates how autophagy controls cisplatin drug response through its effects on molecular pathways and cell death mechanisms in human cancers [20]. Studies demonstrated that autophagy exhibits dual characteristics during tumor progression by describing its role in both cancer formation and immune evasion while making treatment resistant [21]. Guo et al. [22] examine P-glycoprotein (P-gp) functions in drug resistance promotion by studying its relation to regulatory non-coding RNAs and potential nanotechnology-based intervention approaches. The research demonstrates how autophagy functions with drug resistance and tumor survival mechanisms in a complex manner. Medical researchers now use this knowledge to develop new resistance-reducing strategies that enable P-gp- and autophagy-based treatments for cancer patients. Better knowledge of these processes shows promise to revolutionize the medical approach to fighting cancer in the future. This narrative review summarizes recent research on the anticancer potential of pinocembrin, focusing on its molecular mechanisms, interactions with cellular targets SAR, and the role of nanotechnology in improving its therapeutic profile. Literature analysis was carried out on online available servers, including Scopus, PubMed, and Google Scholar, by inserting the keys such as pinocembrin and cancer, apoptosis induction, cell cycle arrest, anti-angiogenesis, and metastasis. In contrast to earlier reviews that have mostly been summaries of the pharmacological and biological actions of pinocembrin [9–11], the current review is a synthesizing and integrative synthesis that relates various levels of evidence to the cancer biology process. Namely, the article is the first to provide the insights of structure–activity relationship (SAR) with detailed mechanistic analysis of the key oncogenic signaling pathways such as PI3K/Akt/mTOR, STAT3, and NF-kB, which play a critical role in tumor progression and resistance to therapy [7, 23]. Notably, the review goes a notch further than descriptive reporting by critically assessing experimental design, active concentrations, model systems, and methodological limitations in all studies which exist thus filling a huge gap in the existing literature. Although there are new aspects, including tumor microenvironment modulation, pharmacokinetic limitations, and resistance-related mechanisms, which are also noted, they are not fully studied, despite the known relevance in cancer treatment [21, 22]. Moreover, although nanotechnology-derived delivery systems have received extensive research on flavonoids, this review outlines the scant but emerging evidence on nanoformulation of pinocembrin without excessive dependence on surrogate compounds. Together, this combined and critical approach makes the current work stand out from the previous reviews and offers a prospective framework for future developments of pinocembrin into clinically relevant anticancer use.

## Chemistry and sources of pinocembrin

Many plant species have been found to contain pinocembrin, including the many genera of the *Piperaceae* family, which includes 14 genera and 1950 species that are said to be a rich source of the compound. Apart from this family, pinocembrin has also been isolated from plants belonging to the numerous species-rich *Lauraceae* and *Asteraceae* families. [24]. A total of 600 species of *Helichrysum* are found in Africa, with roughly 244 of those species occurring in South Africa, while about 250 species of the genus *Cryptocarya* are primarily found in tropical and subtropical climates. [25].

A subclass of flavonoids, pinocembrin (5,7-dihydroxyflavanone) is mostly found in propolis, honey, and a variety of plants, including *Boesenbergia rotunda* and *Penthorum chinense Pursh.* [11]. It has a distinctive chromanone (2-phenylchroman-4-one) nucleus and a basic flavanone backbone made up of a C6-C3-C6 skeleton [26]. Because of its many pharmacological characteristics, such as its anti-inflammatory, anti-cancer, and antioxidant effects, pinocembrin has attracted a lot of research. To improve its medicinal potential, recent research has explored its chemical derivatization and SAR. [10, 27, 28]. Pinocembrin is a prospective neuroprotective agent because of its lipophilic nature, which makes it easier to pass the blood–brain barrier. [29]. Its hydroxyl functional groups scavenge free radicals and inhibit enzymes such as cyclooxygenases (COX), which contribute to its anti-inflammatory and antioxidant properties. Its anti-cancer and antibacterial qualities also depend on its ketone functionality, which is critical for enzyme binding. [30]. Numerous chemical changes, including esterification, glycosylation, and hydroxylation, have been investigated to improve its bioavailability and effectiveness, thereby maximising its medicinal potential. [31].

### Structure–Activity Relationship (SAR) studies

**Fig. 1:**
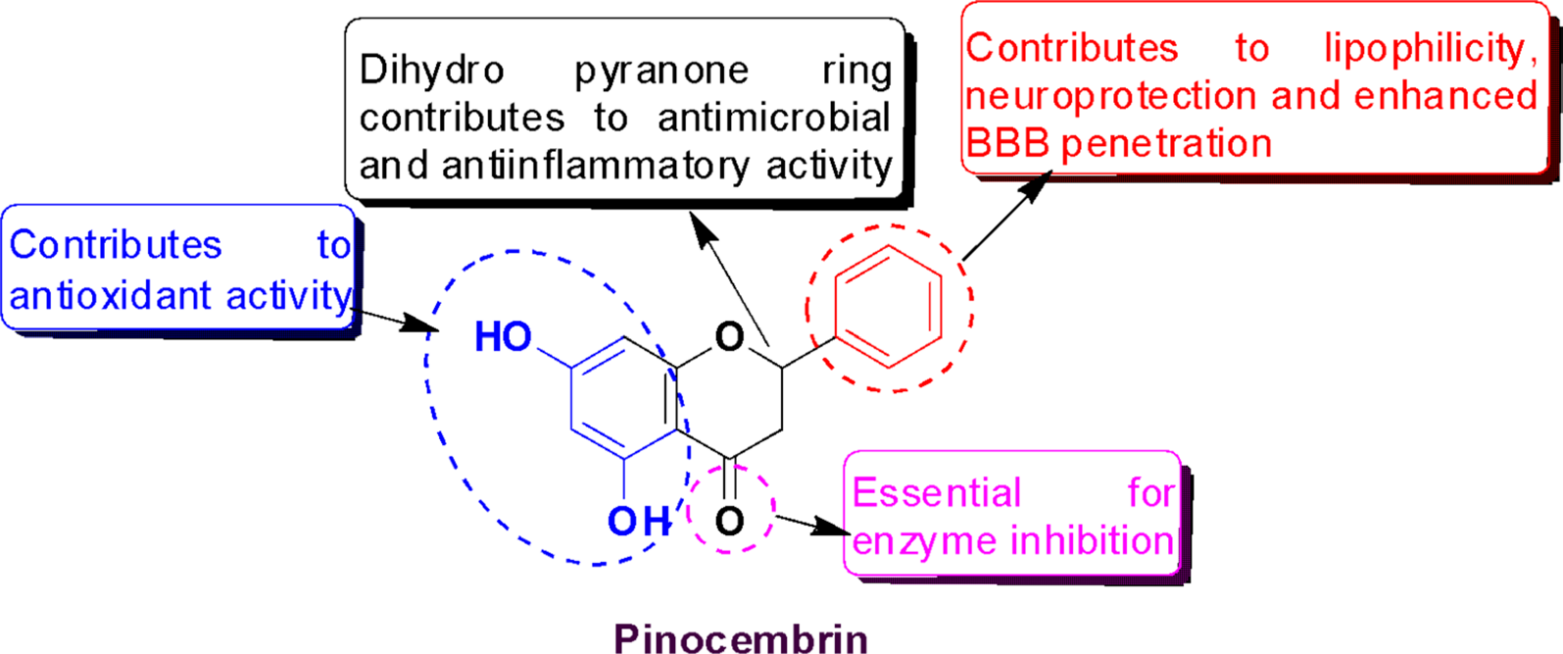
Structural activity relationship of pinocembrin. Its 2,3-dihydro-2-phenylchromen-4-one scaffold and particular hydroxyl substitution pattern directly characterize its chemical reactivity

Optimizing pinocembrin’s biological activity requires an understanding of its SAR. According to a study, fingerroot (*Boesenbergia rotunda*) contains pinocembrin, an α-glucosidase inhibitor and anti-glycation agent [32]. The study examined pinocembrin’s MG-trapping action and suggested potential SARs, indicating that certain structural characteristics may be involved in its ability to suppress the production of advanced glycation end products (AGEs) [33]. Another study used a network pharmacology-based approach to clarify how pinocembrin prevents ovarian cancer. In addition to highlighting the compound’s participation in pathways linked to protein kinase activity and cancer growth, the study identified 163 potential targets [33]. Molecular docking experiments further showed strong binding affinities between pinocembrin and important target proteins, which shed light on its SAR. In order to evaluate possible medication interactions, pinocembrin’s interactions with cytochrome P450 enzymes and drug transporters have also been studied [34]. It was discovered that the substance inhibited specific enzymes and transporters, which may impact its pharmacokinetics and effectiveness. These recent investigations highlight the significance of chemical derivatisation and SAR analyses in augmenting pinocembrin’s medicinal potential [35]. The optimisation of pinocembrin derivatives for a range of clinical applications is still being aided by ongoing research in this field [9]. The structural characteristics that contribute to various biological functions are described in Fig. 1.

The figure displays the essential structural elements of pinocembrin, which enable its biological effects. The hydroxyl groups at positions 5 and 7 enable antioxidant and anti-inflammatory effects because they help scavenge free radicals and block enzymes. The chromanone ring’s ketone group functions as a key binding element, which enables target enzyme and receptor interactions, thus supporting its anticancer and antimicrobial properties. Pinostrobin, naringenin, and other closely related polyphenolic chemicals share the same core structure, largely varying in the degree of substitution (hydroxylation or methoxylation) on their aromatic rings. Variations and chemical modifications such as glycosylation, esterification, and hydroxylation influence bioavailability and therapeutic potency.

## Cellular interactions pinocembrin

### Apoptosis and cell cycle arrest

The scientific community has learnt in the past few decades how powerful natural substances can be in treating terrible illnesses like cancer. Even if there are many different natural bioactive molecules, an efficient cancer treatment is still needed. Therefore, it is crucial to comprehend how these natural chemicals interact with the identified cellular target in order to develop a successful cancer therapy plan. Both intrinsic (mitochondrial) and extrinsic Fas ligand apoptotic cell death in cancer cells are mediated by pinocembrin. The inhibition of inflammatory mediators such as interleukins and Tumor Necrosis Factor-α is another anti-tumor property of pinocembrin. In addition to aiding in the understanding of the biology of cancer, investigating the mechanistic insight into the mode of action of pinocembrin will encourage the scientific community to develop new anti-cancer methods in the future.

A controlled and coordinated cellular process, apoptosis occurs in both healthy and diseased circumstances. Natural cell death in cancer is caused by apoptosis, a closely controlled multi-step process that can be governed by both intrinsic (mitochondrial) and extrinsic (death receptors) mechanisms. During apoptosis, caspases cleave hundreds of proteins to perform both the intrinsic and extrinsic pathways. To modify the cancer microenvironment and find new therapeutic agents, apoptosis may therefore be a promising therapeutic approach to target cellular tumor suppressor mechanisms. [36]. In addition to overcoming the apoptosis-resistant phenotype of the cell, these mechanistic insights may open new doors (Table 1). In fact, the formation of the majority of tumour types is caused by genetic defects that either hyperactivate or suppress the fundamental cellular machineries [37]. Natural phytoconstituents all successfully induce cell-cycle regulation, apoptosis, and anti-proliferative activities. [38].

**Table 1:** An overview of anticancer mechanistic insight of pinocembrin in cancer

S.no	Tumor	Model	Cell lines/ Animal models	Effect	Mechanistic insight	Dose	Ref
1	Breast cancer	In vitro	MCF-7, MDA-MB-231, and SKBR3 cells	Antiproliferative Effect, antimetastatic effect	↓PI3K/AKT, ↓Cdc2 and cyclinB1, ↑BAX, ↓Bcl-2, ↑PARP1, ↑caspase 3/9, ↓survivin	0, 80, 160, or 240 µM	[23]
2	Lung cancer	In vitro	A549 cells	Apoptosis induction, Antiproliferative Effect	↓Ki67 and PCNA, ↑Bax, ↑caspase3 and 9, ↓Bcl-2, ↓LC3-II, ↓Atg5, ↓P62, and ↓Beclin1	25, 50, 100, 150, and 200 µM	[12]
3	Ovarian cancer	In vitro	SKOV3 cells	Apoptotic induction, Antiproliferative Effect	↓N-cadherin, ↓GABAB1 and GABAB2	25, 50, 100, and 200 μM	[15]
4	Colon cancer	In vitro	HCT 116 cells	Apoptotic induction, Antiproliferative Effect	↑Caspase-9, -3, and PARP, ↓Caspase-8, ↑BAX, ↓Bcl-XL	50 -100 μM	[13]
5	Prostate cancer	In vitro	PC-3 cells	Antiproliferative Effect, Apoptotic induction, cell cycle arrest	↑Caspase-9, -3, ↓G0/G1, ↑BAX, ↓Bcl-2	50 -100 μM	[16]
		In vitro	PC3 and DU-145 cells		↓S and G2/M		[36, 54, 55]
6	Colorectal cancer	In vitro	HT29 and HCT116 cells	Antiproliferative Effect, inhibition of migration and Invasiveness	↓MMP-2 and N-cadherin, ↑E-cadherin, and LACTB	50, 100, and 200 μM	[56]
7	Retinoblastoma	In vitro	Y-79 cells	Anti-metastasis, Antiproliferative Effect	↓TGF-β1- vimentin, ↓N-cadherin, ↓αvβ3 integrin, ↓FAK and p38α, ↓MMP-2/-9, ↓ IκBα, ↓NF-κB	0, 5, 10, 20, 40, 60, 80, and 100 μM	[57]
8	Hepatocellular carcinoma	In vitro	HCC cell lines, HepG2 and Li-7 cells	Apoptotic, Cell cycle arrest	↓G1 phase, ↓ STAT3, ↓cyclin D1 and E, ↓CDK4, and CDK6	5 -320 µg/ml	[58]
9	Melanoma	In vitro	B16F10 and A375 cells	Apoptosis induction, Anti-proliferation effect	↑caspase-8, -9 and -3, ↑PARP, ↑GRP78, ↑caspase12 and CHOP, ↑PI3K, ↑AKT and mTOR	100 μM	[17]

**Fig. 2:**
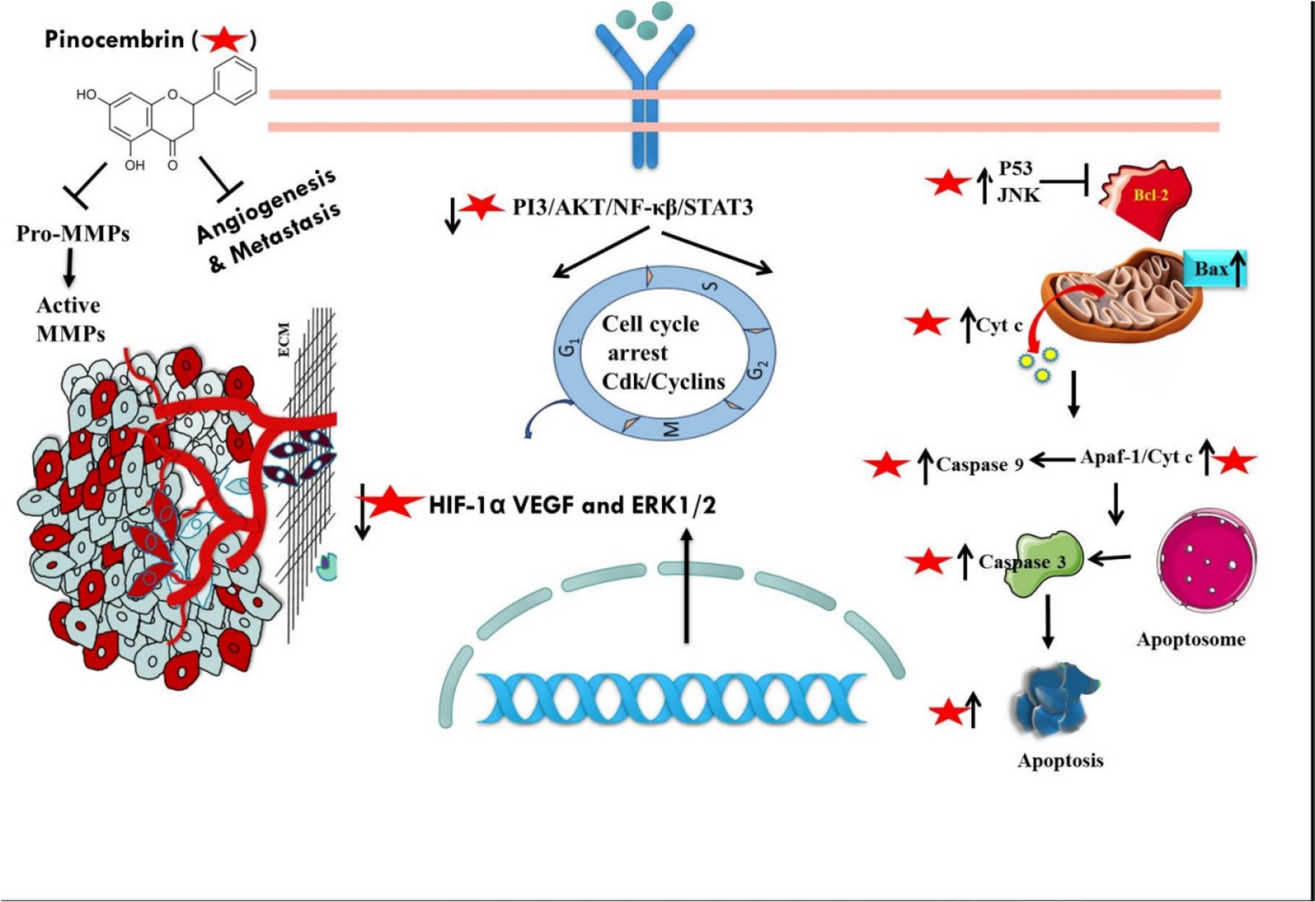
Anticancer mechanisms of pinocembrin: apoptotic and anti-proliferative effects

Pinocembrin (Pino) has been reported to play an important role in the inhibition of cancer development by inducing apoptosis and cell proliferation (Fig. 2). It has specifically been demonstrated to cause cell cycle arrest and apoptosis in colorectal and prostate cancer [39, 40] It can induce apoptosis through multiple pathways, including mitochondria-mediated and endoplasmic reticulum (ER) stress-mediated apoptosis. In colon cancer cell lines, pinocembrin translocates cytosolic BCL2-associated X, Apoptosis Regulator** (**Bax) to mitochondria, causing mitochondria-mediated apoptosis [13]. Similarly, it inhibits autophagy via activating the PI3K/Akt/mTOR mechanistic target of rapamycin pathway and enhances ER stress-induced apoptosis through the IRE1α/Xbp1 pathway in melanoma cell lines [17]. Additionally, it has been shown to inhibit ovarian cancer cells’ migration and proliferation and encourage their apoptosis by downregulating the mRNA levels of the GABAB receptor and N-cadherin [15]. Previous studies have suggested pinocembrin’s role in arresting the cell cycle progression by modulating the levels of kinases (CDKs), too. In human prostate cancer cells, it has potent anticancer effects through G0/G1 cell cycle arrest, endogenous ROS generation, and death [16]. Pinocembrin is also known to inhibit autophagy in a dose-dependent way, which suppresses the growth and promotes apoptosis of A549 lung cancer cells [12]. Furthermore, pinocembrin successfully stopped the growth of prostate LNCaP cells by causing apoptosis and stopping the cell cycle at the S and G2/M stages. The cytotoxic effects are exerted by pinocembrin against colon cancer cell lines (HCT116) with an increase in the expression of caspase-3 and -9, and mitochondrial membrane potential (MMP). Moreover, it also shows relatively less toxicity towards human umbilical cord endothelial cells [9].

### Anti-angiogenic and anti-metastatic properties of pinocembrin

The formation of new blood vessels, or angiogenesis, is a crucial step in the metastatic process. The main pathway for tumor cells to leave the main tumor site and enter the bloodstream is provided by these arteries. Vascular density can serve as a prognostic predictor of metastatic potential for a variety of tumors; initial tumors with high vascular density are more likely to metastasize than those with low vascular density. [9, 41] A key tactic for preventing various solid tumors is to sever or at least reduce the blood supply to tumor microregions, thereby causing pan-hypoxia and pan-necrosis within solid tumor tissues. [42].

Pinocembrin shows its anticancer effects by inhibiting metastatic potential in solid tumors [13, 15, 43]. It has also been reported to suppress TGF-β-induced epithelial-mesenchymal transition (EMT) in human Y-79 retinoblastoma cells by inactivating the αvβ3 integrin/FAK/p38α signalling pathway. In vitro and in vivo studies have observed that pinocembrin suppresses the metastasis of breast cancer cells via regulation of the PI3K/AKT pathway. Moreover, it can also hamper the migration and invasion abilities of MCF-7 and MDA-MB-231 cells at non-cytotoxic concentrations [23]. Additionally, pinocembrin has been shown to down-regulate the production of several metastatic proteins, such as matrix metalloproteases. Angiogenesis also supports metastasis, and pinocembrin’s ability to prevent neovascularisation in the tumor microenvironment is well established. Similarly, it inhibits ovarian cancer cell migration via down-regulating N-cadherin and GABAB receptor mRNA expression [13]. In vitro study also revealed that pinocembrin can modulate HUVECs migration, in vitro organization into capillary-like structures, and ex vivo formation of new blood vessels by affecting HIF1*α*, VEGF, and ERK1/2 signalling pathway [44]. According to Thithuan et al. [45], pinocembrin inhibits the Akt signaling pathway, which lowers MMP production and activity and limits the metastatic potential of HCC cells (HepG2 and Li-7).

The Figure shows how pinocembrin controls different cancer pathways simultaneously. The apoptotic mechanism of pinocembrin functions through intrinsic mitochondrial and extrinsic death receptor pathways to increase caspase-3/9 and pro-apoptotic protein Bax expression while decreasing anti-apoptotic protein B-cell lymphoma 2 (Bcl-2). Pinocembrin reduces cell growth and blocks angiogenesis and EMT processes, and inhibits the expression of Matrix metalloproteinases and N-cadherin metastatic markers. Through blocking the signaling pathways PI3K/Akt/mTOR and Signal Transducer and Activator of Transcription 3 (STAT3), and NF-κB, pinocembrin produces its biological effects.

### Anti-inflammatory and immunomodulation action

Prolonged inflammation can cause cell mutations and foster an environment that is favorable for the growth of cancer. Prostaglandin E-2, cytokines, and cyclooxygenases are examples of inflammatory mediators that are produced in response to inflammation [46]. By preventing the release of these mediators, anti-inflammatory medications are known to reduce the inflammatory response. In a previous study, it was investigated that pinocembrin attenuated LPS-induced lung injury in mouse models through suppression of IκB alpha, c-Jun N-terminal kinase, and p38 mitogen-activated protein kinase activation [47]. In another study, pinocembrin dramatically decreased Th2 cytokines interleukin (IL)-4, IL-5, and IL-13. Additionally, the results showed that in the lung tissue of ovalbumin-sensitized mice, pinocembrin therapy inhibited phosphorylation of inhibitor-κBα (IκBα) and NF-κB subunit p65 activation [48]. Similarly, by preventing NF-κB activation, pinocembrin decreased the levels of Inducible Nitric Oxide Synthase (iNOS), Cyclooxygenase-2, Tumor necrosis factor-α (TNF-α), and Interleukin-1β in *Labeo rohita* head-kidney macrophage [49]. According to Hanieh et al. [50], pinocembrin also decreased TNF-α, IL-6, NO, and iNOS, among other pro-inflammatory mediators in the IgE-mediated allergic response [50]. According to data from Zhou et al. [51], pinocembrin suppressed the PI3K/Akt/NF-κB signaling pathway, which in turn prevented the production of inflammatory mediators generated by LPS.

Improving cancer care is still hampered by the low response rate and significant inter-individual variability in immunotherapy outcomes. According to studies, the histone deacetylase family is essential for controlling tumor immune evasion and immunotherapy sensitivity. [52]. Additionally, Pinocembrin was found to be a specific Histone Deacetylase 7** (**HDAC7) inhibitor that enhances the effectiveness of immunotherapy in BCa by restoring CD8 ^+^ T cell infiltration. Furthermore, by blocking checkpoint inhibitors and cytokines (interleukins and interferons) important in immune cell function, pinocembrin may emerge as a promising immunomodulator. Pinocembrin treatment dramatically decreased ovalbumen-specific IgE in serum and Th2 cytokines interleukin (IL)-4, IL-5, and IL-13 in bronchoalveolar lavage fluid. [48]. Similarly, Darwish et al. [53] examined pinocembrin’s unique hepatoprotective qualities against cisplatin hepatotoxicity by focusing on apoptosis, oxidative stress, and the interaction between mitogen-activated protein kinase kinase kinase 7 and the inflammatory cascade. [53].

## Pinocembrin as a multi-target anticancer agent: an integrative view

Pinocembrin is a promising multi-target anticancer flavanone due to its capacity to control several cancer hallmarks, which are uncontrolled cell proliferation, anti-apoptotic effects, metastasis, angiogenesis, inflammation, and redox imbalance. There is also recent preclinical data that supports its anticancer application by showing that pinocembrin functions on important oncogenic signalling cascades that are often dysregulated in cancerous cells.

Pinocembrin has a strong antiproliferative effect at the molecular level by altering the PI3K/Akt/mTOR, STAT3, and NF-kB pathways, which prompts the inhibition of the survival signaling pathway and reinstates apoptotic sensitivity. Recent in vitro and in vivo experimental results have indicated that pinocembrin causes cell-cycle arrest in the G0/G1 or G2/M phases by suppressing the cyclins (cyclin D1, cyclin B1) and the cyclin-dependent kinases (CDK4/ CDK6), thereby restraining the growth of tumor cells. In models of hepatocellular and colorectal cancer, pinocembrin has been demonstrated to have a powerful protective effect against clonogenic survival and proliferative capacity and no impact on normal cells, which demonstrates its selective anticancer properties. [58].

Besides growth inhibition, pinocembrin is also capable of inducing programmed cell death by involving intrinsic and extrinsic apoptotic pathways. The depolarization of mitochondrial membrane, the translocation of Bax, cytochrome-c release, and caspase-3 and caspase-9 activation have been reliably identified in various cancerous cell types. A more profound preclinical study also recently revealed that pinocembrin inhibits anti-apoptotic proteins, including Bcl-2 and survivin, and thus overcomes apoptosis resistance, one of the most prominent barriers to cancer treatment [7].

Pinocembrin also exhibits moderate anti-metastatic and anti-invasive effects due to its ability to affect EMT and extracellular matrix remodelling. Recent experiments on colorectal cancer showed that pinocembrin significantly suppresses the invasion and mobility of cancer cells and reverses the down-regulation of mesenchymal (N-cadherin, vimentin) and matrix metalloproteinases (MMP-2 and MMP-9) and epithelial (E-cadherin) markers. [58]. The results indicate that pinocembrin disrupts metastatic spread, other than simply having cytotoxic effects.

Moreover, pinocembrin is important in the inhibition of angiogenesis and inflammation of tumors, which are two processes that are interrelated and facilitate tumor development. Angiogenic mediators such as Vascular Endothelial Growth Factor (VEGF) and Hypoxia-inducible factor-1alpha have also been reported to be suppressed by pinocembrin in preclinical breast cancer models, leading to inhibited neovascularization and tumor progression [23]. Its anti-inflammatory effect, which operates via NF-KB signaling and silencing of inflammatory cytokines like TNF-alpha and IL-6, also plays a role in the interference of the tumor-promoting microenvironment.

Put together, this body of new evidence supports the use of pinocembrin as a multi-functional anticancer agent with the ability to inhibit tumor growth, survival, invasion, angiogenesis, and inflammation at the same time. In spite of the fact that most of the evidence is still preclinical, the combination of these mechanisms (backed by recent 2024–2025 research) contributes to the potential of pinocembrin to be used as an adjunctive therapeutic or chemopreventive agent. However, additional in vivo confirmation, pharmacokinetic characterization, and well-structured clinical trials are needed to give full descriptions of its translational applicability in cancer treatment.

## In vivo anticancer evidence of pinocembrin

Although most of the research on the anticancer properties of pinocembrin has focused on in vitro experiments, there is also a growing body of in vivo evidence that is essential in providing proof of its biological applicability and potential in treating cancer. The animal studies reported in support of these assertions indicate that pinocembrin may be used to regulate the growth and progression of tumors in complex biological systems with a good safety profile. Punvittayagul et al. [43] indicated that in a chemically induced rat hepatocarcinogenesis model, pinocembrin had a significant antitumor initiation and promotion effect. The pinocembrin treatment led to a significant decrease in the preneoplastic lesions of the liver, oxidative stress inhibition, and inhibition of the inflammatory reaction in the liver tissues. Notably, these effects were seen despite the absence of an apparent systemic toxicity, and this suggests that pinocembrin might serve as a chemoprevention agent by disrupting early oncogenic pathways, as opposed to producing an unselective cytotoxic effect. The antitumor efficacy of pinocembrin in vivo is further proven by complementary evidence provided by tumor xenograft models [59]. Zhu et al. [23] found that pinocembrin treatment markedly reduced tumor growth, as well as the expression of proliferative and angiogenic markers, including the elements of the PI3K/AKT signaling pathway and VEGF, in a mouse model of breast cancer. It is important to note that no major changes in body weight, evidence of liver or kidney impairment, or histopathological destruction of major organs were observed in treated animals, and this demonstrates that the compound is tolerable in vivo. In addition to tumor growth inhibitory effects, in vivo evidence indicates pinocembrin can regulate tumor-related angiogenesis and metastatic ability in an indirect manner by regulating hypoxia- and inflammation-mediated pathways, instead of directly by vascular destruction. These are in line with its reported capacity to control signaling pathways related to epithelial-mesenchymal transition and extracellular matrix remodeling in preclinical conditions. Although these results are encouraging, the existing in vivo research is so far both limited in its scope and heterogeneous, due to cancer type, dose schedule, and experimental design. The majority of studies do not include thorough pharmacokinetic analysis, dose response studies, and long-term outcome [60]. Therefore, although the translational relevance of the anticancer effects of pinocembrin is supported by available animal data, in vivo studies with approved biomarkers, preclinically relevant model tumors, and high-quality toxicological assessment are needed to further develop the evidence database and enable the transition to clinical application.

## Synergistic and nanotechnology-mediated perspectives

The clinical applications of pinocembrin encounter hurdles due to its high-water insolubility and decreased oral bioavailability, as well as rapid degradation rates in the body. Nanotechnology-based drug delivery systems function as an effective strategy to improve the therapeutic value of natural compounds, including pinocembrin, by overcoming current delivery obstacles. The delivery of pinocembrin becomes more effective through nanocarrier systems such as polymeric nanoparticles, liposomes, nanoemulsions, and micelles because these systems enhance solubility, protect the compound from breakdown, provide extended drug release, and direct the compound to cancer cells [59]. This section reviews different nanotechnology delivery methods that enhance preclinical pinocembrin research. By reducing the dosage of medicines, synergistic therapy can prevent potential side effects during treatment. Research has demonstrated that various phenolic compound combinations, such as pinocembrin, can have hormetic effects on non-tumorogenic cells and synergistic lethal effects on breast cancer [60]. Simvastatin and pinocembrin have also been shown to have protective benefits in Apolipoprotein E − / − mice with hyperlipidemia [61]. Current research has noted the encouraging potential of natural compounds together with innovative technological approaches in cancer management and prevention. The monoterpenoid phenol compound carvacrol causes human prostate cancer cells to undergo programmed cell death while halting cell cycle progression through Notch signaling inhibition [62]. The chemopreventive properties of flavonoids as plant-derived polyphenols are well-established because these compounds regulate signaling pathways that control cell proliferation and apoptosis [63].

Research shows that Intricatinol works better with cisplatin to treat cancer cells by activating the p38 MAPK/p53 signaling pathway in A549 lung cancer cells. [64]. The PI3K/Akt/mTOR pathway is a vital tumor growth and survival regulator, making flavonoids promising therapeutic candidates. [65]. Nanotechnology serves as a powerful cancer chemoprevention tool because it enables targeted drug delivery systems that improve drug availability. [66].

Research in patent literature demonstrates the therapeutic value of polyphenols while validating their anticancer properties for future drug development. [67]. Modern oncology benefits from natural compounds and innovative delivery systems that were demonstrated through these recently discovered findings. In addition, hydrophobic substances like pinocembrin can have their pharmacokinetic profile enhanced by nanoformulations such as nanoparticles, liposomes, and nanoemulsions. These formulations seek to improve pinocembrin’s solubility, shield it from deterioration, and enable regulated release by encasing it in nanocarriers, thus boosting its therapeutic effectiveness. Research has shown that pinocembrin-loaded nanoparticles had better stability and bioavailability compared to free pinocembrin. In models of cerebral ischemia, for example, pinocembrin-loaded nanoparticles have improved neuroprotective effects, indicating possible therapeutic uses in the treatment of ischaemic stroke. [44].

In another investigation, researchers have used F127/MPEG-PDLLA to create polymeric micelles filled with pinocembrin. The entrapment effectiveness of these micelles was 90.53%, and their particle size was roughly 27.63 nm. Studies on in vivo pharmacokinetics showed that oral bioavailability was 5.3 times higher than that of free pinocembrin [59]. It has been demonstrated that the formation of a complex with lecithin greatly increases the solubility of pinocembrin in n-octane and water. This complexation increases antioxidant activity and improves solubility, indicating possible uses in clinical and medical contexts. [20] Table 2.

**Table 2:** Nanotechnology-based delivery systems enhancing the therapeutic potential of flavonoid compounds

Flavonoid compound	Nanotechnology used	Cancer model / Application	Key outcomes	Reference
Quercetin	Polymeric nanoparticles	TNBC cells (MDA-MB231 cells)	Improved bioavailability and targeted delivery; enhanced apoptosis and efficacy	[30]
Baicalein	PCL-PEG nanoparticles	Colorectal cancer (HCT116 and HT29 cells)	Enhanced cytotoxicity and sustained drug release	[68]
Epigallocatechin-3-gallate (EGCG)	Lipid-based nanocarriers	Breast cancer (MCF-7, MDA-MB-231, and MCF-7TAM)	Increased bioavailability; improved therapeutic index	[69]
Curcumin	(CUR)- loaded poly (lactic-glycolic acid) nanoparticles (PLGA-CUR NPs)	Prostate cancer (LNCaP)	IκBα Overexpression	[70, 71]
Naringenin	Chitosan nanoparticles	Breast cancer (MCF-7 cell)	Increased P53,NFκB	[72]
Apigenin	Lipid nanoparticles	Colon cancer (HCT-116)	Improved bioavailability, apoptosis, and cell cycle arrest	[73]

## Safety aspects

The safety of pinocembrin is as crucial as its efficacy. In a randomized, double-blind, placebo-controlled clinical trial with 58 healthy Chinese volunteers, demonstrated a good tolerability of this phytochemical, administered as a single ascending dose (20–150 mg) through the intravenous route. No serious adverse events or discontinuations were described in this study. The maximal plasma concentrations remained in the low micromolar range (up to 2.46 µg/ml) with no evidence of accumulation. [74]. However, further safety studies on the oral administration and chronic treatment of pinocembrin are still highly required, both in healthy subjects and patients with different diagnoses.

## Future prospects and research directions

Although there is a considerable amount of preclinical data on the anticancer potential of pinocembrin, there are a number of important gaps that have to be bridged before its effective clinical translation. More focused future studies are needed to demonstrate the robust in vivo validation of the findings with clinically relevant tumor models, such as orthotopic and patient-derived xenografts, to improve the representation of tumor heterogeneity and complexity of the microenvironment. There is a particular need to establish therapeutic windows and determine long-term efficacy with the help of standardized dose–response studies and long-term treatment regimens. One of the key directions of the future is the optimization of various pharmacokinetic and bioavailability factors, as the low water solubility and high rate of pinocembrin metabolism lead to decreased systemic exposure of the compound at the moment. It should be further developed in terms of advanced formulation strategies; in particular, polymeric nanoparticles, lipid-based carriers, and targeted nano-systems, which could enhance stability, tumor accumulation, and controlled release. Structural comparisons of free and nano-encapsulated pinocembrin, on the same experimental models, would give invaluable information about formulation-dependent therapeutic benefit. The other prospective area is the incorporation of pinocembrin into combination therapy regimes. Since pinocembrin can be used to multi-targetedly modulate oncogenic signaling, inflammation, and oxidative stress, this compound can be beneficial in potentiating the effect of conventional chemotherapeutics or the targeted agent, and may also decrease dose-related toxicity. Systematic research on synergistic interactions, the most effective sequencing protocols, and resistance-modulating in both in vitro and in vivo systems should be conducted in the future.

Mechanistically, one would expect to understand more about context-dependent responses by delving into them further. The anticancer action of pinocembrin seems to be homogeneous across different types and molecular subtypes of cancer, which highlights the importance of using biomarkers to select responsive patient groups. The less developed directions, such as cancer metabolism, tumor immunomodulation, and microbiome-drug interactions, are also under-researched but potentially effective areas of investigation for pinocembrin.

Lastly, clinical translation is still in its early years, and there is a lack of human safety and pharmacokinetic information. Well-planned initial clinical trials are necessary to determine tolerability, the best route of administration, and initial signs of efficacy. The possibility of regulatory issues, the ability to source natural compounds, and formulate pinocembrin in large quantities will also be addressed to ascertain the feasibility of pinocembrin as a clinically applicable anticancer agent.

In short, pinocembrin is a promising multi-functional phytochemical with a high potential in the management of cancer, and its future success will require a harmonious development of the formulation science, translational pharmacology, and precision-focused preclinical and clinical research.

## Conclusion

The review identifies the anticancer properties of flavanone pinocembrin while highlighting its multiple effects against cancer through apoptosis induction, together with cell cycle arrest, along with anti-inflammatory properties and angiogenesis inhibition mechanism, as well as metastatic pathway suppression. Multiple preclinical studies involving breast cancer, colon cancer, prostate cancer, lung cancer, and ovarian cancer showed that pinocembrin regulates the PI3K/Akt and STAT3 and NF-κB signaling pathways. The study of structure–activity relationships through chemical modifications provides researchers with potential methods to improve both biological activity and selectivity of the compound. The development of nanotechnology-based delivery systems has brought better pharmacokinetic performance to pinocembrin through improved stability, along with enhanced solubility and tumor-targeted delivery capabilities. As a result, these strategies both boost pinocembrin’s therapeutic features and decrease unintended effects, therefore increasing its development potential for medical applications. The clinical data indicate that pinocembrin functions as either an additional treatment or combination therapy in cancer care by fighting drug resistance and reducing conventional chemotherapy side effects. The low toxicity levels toward regular cells, combined with its synergistic interactions with conventional chemotherapeutic drugs, make it suitable for translation into clinical practice. Multiple research gaps need resolution before clinical deployment because scientists require extensive safety data about long-term use, along with studies of oral absorption and proper dosage methods, and drug interaction analysis. Future research needs to conduct well-designed clinical trials for evaluating both the effectiveness and tolerability of pinocembrin when used to treat cancer patients and its potential in personalized medicine strategies. The natural foundation of pinocembrin, together with its multiple mechanisms of action and positive findings in safety assessments, positions it to play an effective role in current oncology therapeutic plans.

## Data Availability

No datasets were generated or analysed during the current study.
